# Semi-Supervised Projective Non-Negative Matrix Factorization for Cancer Classification

**DOI:** 10.1371/journal.pone.0138814

**Published:** 2015-09-22

**Authors:** Xiang Zhang, Naiyang Guan, Zhilong Jia, Xiaogang Qiu, Zhigang Luo

**Affiliations:** 1 College of Computer, National University of Defense Technology, Changsha 410073, China; 2 National Laboratory for Parallel and Distributed Processing, National University of Defense Technology, Changsha 410073, China; 3 Department of Chemistry and Biology, College of Science, National University of Defense Technology, Changsha, Hunan, China; 4 College of Information System and Management, National University of Defense Technology, Changsha, Hunan, 410073 China; University of Memphis, UNITED STATES

## Abstract

Advances in DNA microarray technologies have made gene expression profiles a significant candidate in identifying different types of cancers. Traditional learning-based cancer identification methods utilize labeled samples to train a classifier, but they are inconvenient for practical application because labels are quite expensive in the clinical cancer research community. This paper proposes a semi-supervised projective non-negative matrix factorization method (Semi-PNMF) to learn an effective classifier from both labeled and unlabeled samples, thus boosting subsequent cancer classification performance. In particular, Semi-PNMF jointly learns a non-negative subspace from concatenated labeled and unlabeled samples and indicates classes by the positions of the maximum entries of their coefficients. Because Semi-PNMF incorporates statistical information from the large volume of unlabeled samples in the learned subspace, it can learn more representative subspaces and boost classification performance. We developed a multiplicative update rule (MUR) to optimize Semi-PNMF and proved its convergence. The experimental results of cancer classification for two multiclass cancer gene expression profile datasets show that Semi-PNMF outperforms the representative methods.

## Introduction

In cancer prognosis and treatment, it is crucial to identify different cancer types and subtypes. Traditional methods often rely on similar morphological appearances but easily induce different clinical courses and responses to therapy because of subjective interpretations and personal experience. This usually results in diagnostic confusion. Fortunately, the emergence of the DNA microarray technique removes this barrier in an objective and systematic manner and has showed great potential in outcome prediction of cancer types in genome-wide scales [1–11].

Numerous learning methods have been developed for cancer classification based on gene expression profiles [1–3]. For instance, Golub *et al.* [1] used a weighted voting scheme for the molecular classification of acute leukemia. Nguyen *et al.* [3] incorporated partial least squares (PLS) into the logistic discrimination and quadratic discriminant analysis for tumor classification. However, these methods are not convenient for practical applications because the labeled samples are quite expensive in the clinical cancer research community. To overcome this deficiency, Xu *et al.* [12] used the semi-supervised Ellipsoid ARTMAP (ssEAM) method for cancer classification. Shi *et al.* [13] utilized the semi-supervised method termed low density separation (LDS, [14]) to classify different types of cancers. Moreover, Maulik *et al.* [15] investigated the effectiveness of transductive SVM (TSVM, [16]) in cancer classification. Nevertheless, these algorithmic challenges involve the curse of dimensionality, which indicates that the overwhelming number of measures for gene expression levels contrast with the small number of samples.

This problem often calls for dimension reduction techniques. This paper focuses on non-negative matrix factorization (NMF, [17, 18]) because it is a flexible framework for conducting dimension reduction and performing classification and clustering tasks [19–26]. NMF decomposes a data matrix into the product of two non-negative factors. Due to its effectiveness, NMF and its variants have been applied to analyzing large-scale gene expression datasets [27–29], cancer classification [30, 31] and new class discovery [30]. Brunet *et al.* [31] originally adopted NMF to uncover molecular meta-patterns by clustering samples of leukemia, medulloblastoma and central nervous system tumors, and indicating that NMF outperforms both hierarchy clustering (HC) and self-organizing map (SOM). However, NMF does not explicitly guarantee the sparseness of the decomposition and violates the uniqueness property. Recent works [32] show that this often degrades the clustering performance. To address this issue, Li *et al.* [32] proposed local NMF (LNMF) to overcome this deficiency by imposing the sparse constraints over the decomposition. Hoyer *et al.* proposed sparse NMF (SNMF, [33]) to enforce sparseness in NMF by penalizing the number of non-zero entries of the coefficients rather than the sum of the entries. Furthermore, Gao *et al.* [34] utilized SNMF to identify the meta-patterns of various cancers for identifying different types of tumors.

Because the aforementioned methods follow regularization theory, they are jointly non-convex and are difficult to optimize. Unlike the above methods, Yuan *et al.* [35] developed the projective NMF (PNMF) to induce parts-based representation by implicitly imposing the orthogonal constraint over the basis. However, because these methods are unsupervised learning methods that do not take into account labels, their performance in cancer classification can be further improved. In this paper, we propose a semi-supervised projective NMF method (Semi-PNMF) that utilizes both labeled and unlabeled samples to boost classification performance. Particularly, Semi-PNMF learns a non-negative subspace from concatenated labeled and unlabeled samples and predicts classes by the index of the largest entries of their coefficients. Benefiting from the unlabeled data, Semi-PNMF can learn more representative subspaces, which are beneficial for classification tasks. We explored a multiplicative update rule (MUR) to solve Semi-PNMF and proved its convergence. The experimental results of cancer identification for multiclass cancer gene expression profile datasets including GCM [8] and Acute Leukemia [36] datasets show that Semi-PNMF outperforms the representative methods in terms of quantity.

## Materials and Methods

### Semi-supervised Projective Nonnegative Matrix Factorization

Projective non-negative matrix factorization (PNMF) learns a non-negative projection matrix to project high-dimensional data into the lower-dimensional subspace. Because it can learn parts-based representation, PNMF has been widely applied in pattern recognition [21, 26, 35, 37]. Here, we introduce the other representation form of PNMF that learns the lower-dimensional coefficients of samples to approximate the class indicator for clustering. This is based on the assumption that the basis lies in the subspace spanned by the original samples. Given the data matrix *V* = [*v*_1_,⋯,*v*_*n*_]^*T*^ ∈ *R* ^*n* × *m*^, where *n* denotes the number of samples and *m* their dimensionality, PNMF learns the coefficients *H* ∈ *R* ^*n* × *r*^ to represent original samples, i.e.,

$$\begin{array}{c} \min_{H\geq 0}{∥V-HH^{T}V∥}_{F}^{2}, \end{array}$$ (1)

where ∥•∥_*F*_ denotes the matrix Frobenius norm and *r* the number of clusters.

As in objective (1), it is non-trivial to analyze the convergence in theory because Eq (1) contains a fourth-order term. To remove such a high order term, we first introduce an auxiliary variable, i.e., the cluster centroids, and the equality constraint into Eq (1). Thus, we can obtain

$$\begin{array}{c} \begin{array}{c} \min_{H\geq 0}{∥V-HW∥}_{F}^{2},\\ s.t.,W=H^{T}V. \end{array} \end{array}$$ (2)

The objective is very similar to BPNMF [26], but we cannot directly apply the optimization algorithm of BPNMF to optimize it especially when additional constraints such as the sparseness constraint and Laplacian regularization are imposed over the coefficients, as these constraints easily induce PNMF to produce the trivial solution. To avoid such a drawback, we propose a semi-supervised PNMF method (Semi-PNMF) by recasting Eq (2) as

$$\begin{array}{c} \min_{H,W\geq 0}\frac{1}{2}{∥V-HW∥}_{F}^{2}+\frac{\alpha }{2}{∥W-H^{T}V∥}_{F}^{2}, \end{array}$$ (3)

where *α* ≥ 0 is a regularization constant and *W* denotes the non-negative cluster centroid. Model (3) significantly differs from BPNMF because Eq (3) favors the representative capacity of the cluster centroids, while BPNMF focuses on the orthogonality of the non-negative subspace. Thus, Eq (3) induces the sparse coefficients, while BPNMF produces the sparse basis.

According to Eq (3), we can incorporate the local coordinate constraint [38] to improve the representative power of the basis, meanwhile further inducing the sparse coefficients to be true classes. Thus, we recast Eq (3) as the following regularization form:

$$\begin{array}{c} \min_{H,W\geq 0}\frac{1}{2}{∥V-HW∥}_{F}^{2}+\frac{\alpha }{2}{∥W-H^{T}V∥}_{F}^{2}+\frac{\beta }{2}\sum_{i=1}^{n}\sum_{j=1}^{r}|H^{ij}|{∥V^{i}-W^{j}∥}_{2}^{2}, \end{array}$$ (4)

where *β* trades off the local coordinate regularization and *H* ^*ij*^ denotes the *i*-the row and *j*-th column element of coefficients *H*, *W* ^*j*^ and *V* ^*i*^, signifying the *i*-th and *j*-th row vector of *W* and *V*, respectively.

To make full use of partial labeled samples, we propagate the labels of labeled samples to unlabeled ones by minimizing the distance between their coefficients and the corresponding class indicator. Particularly, we require the coefficients of labeled samples to be equivalent with the corresponding class indicator. Consider the first *d* examples labeled and the rest unlabeled; the data matrix *V* can be divided into two parts, i.e., $V={\left[V_{L}^{T},V_{U}^{T}\right]}^{T}$. Then, we can obtain the objective function of Semi-PNMF as follows:

$$\begin{array}{c} \min_{W,H_{U}\geq 0}J=\frac{1}{2}{∥[\begin{array}{c} V_{L}\\ V_{U} \end{array}]-[\begin{array}{c} Q\\ H_{U} \end{array}]W∥}_{F}^{2}+\frac{\alpha }{2}{∥W-H_{U}^{T}V_{U}∥}_{F}^{2}+\frac{\beta }{2}\sum_{i=1}^{n_{U}}\sum_{j=1}^{r}|H_{U}^{ij}|{∥V_{U}^{i}-W^{j}∥}_{2}^{2}, \end{array}$$ (5)

where *Q* denotes the partial label matrix wherein *Q*_*ij*_ = 1 if *v*_*i*_ belongs to the *j*-th class; otherwise, *Q*_*ij*_ = 0. Both *H*_*U*_ and *n*_*U*_ denote the coefficients and number of the unlabeled samples, respectively.

**Fig 1:**
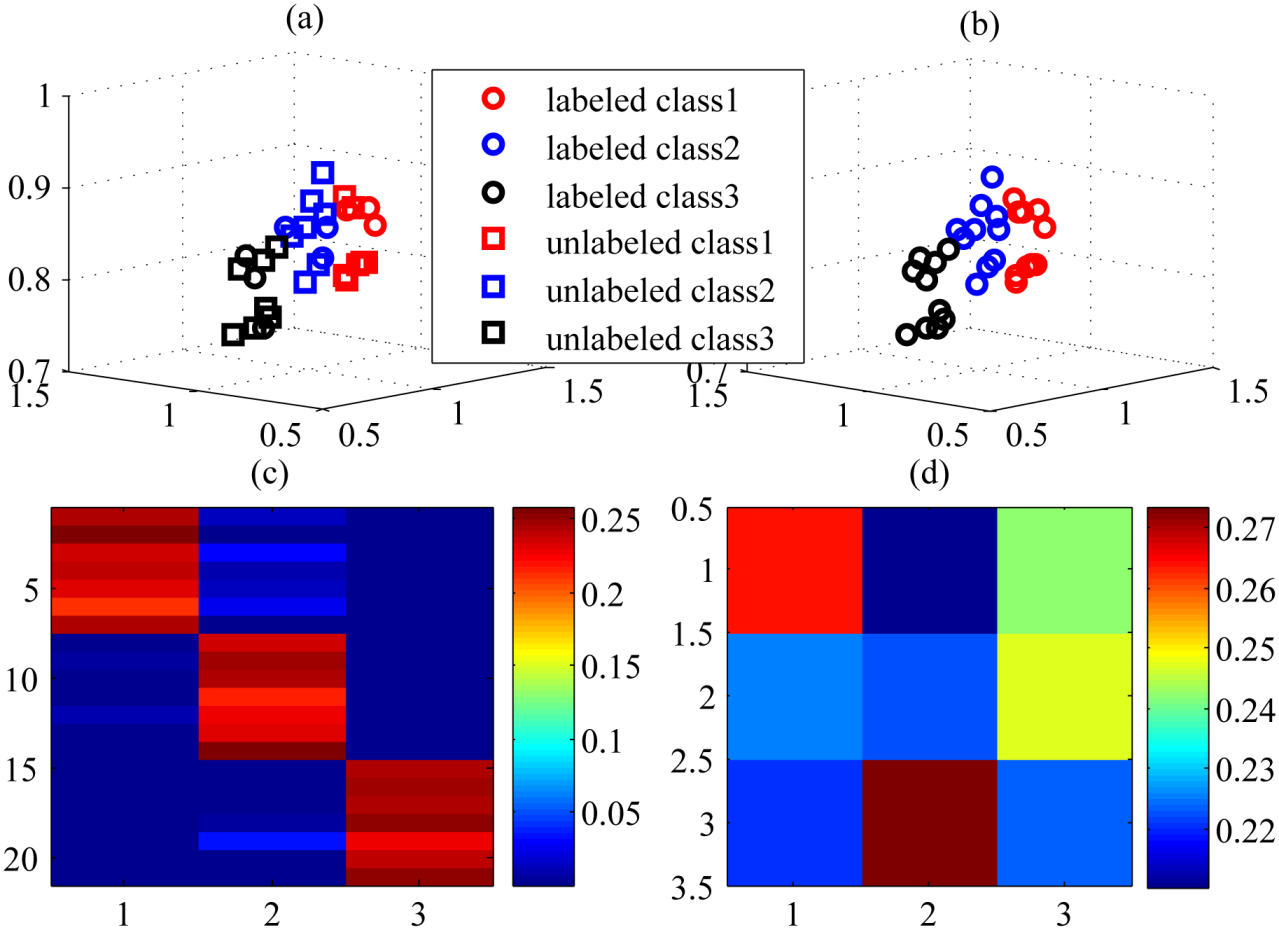
The toy example illustrating (a) the synthetic 3D original data including the labeled and unlabeled samples and the ground-truth labels, (b) the labeled results of unlabeled samples, (c) the learned coefficients of the unlabeled samples, and (d) the learned basis by Semi-PNMF. In Fig (a), both the square and circle markers signify the unlabeled and labeled samples, respectively, and three different colors stand for three different categories. Fig (b) shows that the unlabeled samples are marked as the ground-truth markers and colors. Figs (c) and (d) shows the coefficients and basis learned by Semi-PNMF, respectively. The index of maximum value of the coefficient for an unlabeled sample appears in red and indicates its class.

**Fig 2:**
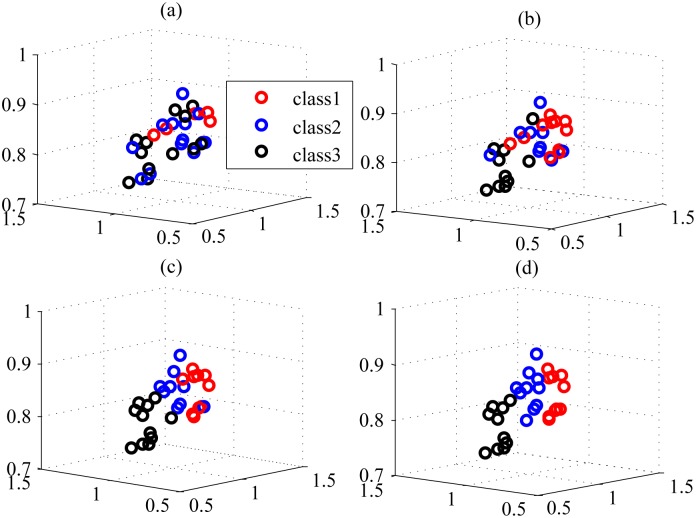
The toy example illustrating the labeling results obtained from the coefficients when the propagation procedure arrives at the (a) initialization stage, (b) 50-th iteration round, (c) 300-th iteration round, and (d) the resultant convergence (1500-th iteration round), respectively.

Interestingly, Semi-PNMF has two distinct aspects. First, it replaces the learned coefficients of the labeled samples with the corresponding class indicator. The constraint is so strong that the learned basis completely biases the labeled samples. This might induce the trivial solution to the coefficients of the unlabeled samples. Second, Semi-PNMF completely ignores the representation contribution of the labeled samples. It is so unintelligible that the learned basis only favors the unlabeled samples. It appeared that both aspects contradict each other, but intrinsically, they mutually complement each other in our Semi-PNMF. In essence, the first aspect corresponds to supervised learning, which generates the reasonable solution yet does not ensure it is consistent with the underlying data distribution, while the second one considers data distribution but cannot yield the reasonable solution. Thus, the combination of both aspects can mutually complement each other. Semi-PNMF learns the shared basis by the labeled and unlabeled instances, meanwhile inducing similar instances to have a similar representation, i.e., the coefficients. Because we impose the restriction that coefficients of the labeled samples be their labels as well as the local coordinate constraint over the basis and coefficients, the unlabeled sample coefficients are implicitly as sparse as the label vectors. In this way, Semi-PNMF effectively propagates the labels of labeled samples to the unlabeled ones. Consequently, in cancer classification, it is reasonable that, for each unlabeled sample, we choose the index of the largest entry of its coefficient to predict the classes of this sample once objective (5) yields their coefficients. The above intuition can be further verified by the toy example given in Figs 1 and 2.

### Optimization Algorithm

It is difficult to optimize Eq (5) because it is jointly non-convex with respect to both *W* and *H*. Fortunately, it is convex with respect to *W* and *H*, respectively. Thus, we can establish the following theorem:

**Theorem 1:** The objective function (5) is non-increasing under the following multiplicative update rules:

$$\begin{array}{c} W=W⊗\frac{Q^{T}V_{L}+\left(1+\alpha +\beta \right)H_{U}^{T}V_{U}}{Q^{T}QW+H_{U}^{T}H_{U}W+\alpha W+\beta F_{U}W}, \end{array}$$ (6),

and

$$\begin{array}{c} H_{U}=H_{U}⊗\frac{\left(1+\alpha +\beta \right)V_{U}W^{T}}{H_{U}WW^{T}+\alpha V_{U}V_{U}^{T}H_{U}+\beta /2\left(A+B\right)}, \end{array}$$ (7)

where ⊗ denotes the element-wise product operator, *F*_*U*_ = *diag*(*sum*(*H*_*U*_)), *A* = [*a*,⋯,*a*] wherein $a=diag(V_{U}V_{U}^{T})$, and *B* = [*b*,⋯,*b*], wherein *b* = *diag*(*WW* ^*T*^).

**Proof.** According to Eq (5), we can obtain the objective with respect to *W* as follows:

$$\begin{array}{c} \begin{array}{c} J\left(W\right)=\frac{1}{2}Tr\left(-2V_{L}W^{T}Q^{T}+QWW^{T}Q^{T}\right)+\frac{1}{2}Tr\left(-2V_{U}W^{T}H_{U}^{T}+H_{U}WW^{T}H_{U}^{T}\right)\\ +\frac{\alpha }{2}Tr\left(WW^{T}-2WV_{U}^{T}H_{U}\right)+\frac{\beta }{2}Tr\left(\sum_{i=1}^{n_{U}}{\left(V_{U}^{i}\right)}^{T}1^{T}\Lambda_{i}1V_{U}^{i}-2W^{T}H_{U}^{T}V_{U}+W^{T}F_{U}W\right) \end{array}, \end{array}$$ (8)

where $\Lambda_{U}^{i}$ denotes the diagonal matrix whose diagonal elements are the *i*-th row vector values of *V*_*U*_.

By Eq (8), we can define the auxiliary function of *J*(*W*) as

$$\begin{array}{c} \begin{array}{c} G\left(W,W^{\prime }\right)=-\left(1+\alpha \right)Tr\left(WV_{U}^{T}H_{U}\right)-Tr\left(WV_{L}^{T}Q\right)\\ +\frac{1}{2}\sum_{ij}\frac{{\left(Q^{T}QW^{\prime }\right)}_{ij}}{{W^{\prime }}_{ij}}W_{ij}^{2}+\frac{1}{2}\sum_{ij}\frac{{\left(H_{U}^{T}H_{U}W^{\prime }\right)}_{ij}}{{W^{\prime }}_{ij}}W_{ij}^{2}+\frac{\alpha }{2}Tr\left(WW^{T}\right)\\ -\beta Tr\left(W^{T}H_{U}^{T}V_{U}\right)+\frac{\beta }{2}\sum_{ij}\frac{{\left(F_{U}W^{\prime }\right)}_{ij}}{{W^{\prime }}_{ij}}W_{ij}^{2}. \end{array} \end{array}$$ (9)

Obviously, objective (9) has

$$\begin{array}{c} G\left(W,W^{\prime }\right)\geq J\left(W\right)=G\left(W,W\right). \end{array}$$ (10)

We can obtain the derivative of Eq (9) as follows:

$$\begin{array}{c} \begin{array}{c} \frac{\partial G(W,W^{\prime })}{\partial W_{ij}}=-{\left(\left(1+\alpha \right)H_{U}^{T}V_{U}-Q^{T}V_{L}\right)}_{ij}+\frac{{\left(Q^{T}QW^{\prime }\right)}_{ij}}{{W^{\prime }}_{ij}}W_{ij}\\ +\frac{{\left(H_{U}^{T}H_{U}W^{\prime }\right)}_{ij}}{{W^{\prime }}_{ij}}W_{ij}+\alpha W_{ij}-\beta {\left(H_{U}^{T}V_{U}\right)}_{ij}+\beta \frac{{\left(F_{U}W^{\prime }\right)}_{ij}}{{W^{\prime }}_{ij}}W_{ij}, \end{array} \end{array}$$ (11)

Based on Eq (11), we have

$$\begin{array}{c} W_{ij}=W_{ij}^{\prime }\frac{{\left(Q^{T}V_{L}+\left(1+\alpha +\beta \right)H_{U}^{T}V_{U}\right)}_{ij}}{{\left(Q^{T}QW^{\prime }+H_{U}^{T}H_{U}W^{\prime }+\alpha W^{\prime }+\beta F_{U}W^{\prime }\right)}_{ij}}. \end{array}$$ (12)

By simple algebra, the formula (6) can be deduced from Eq (12). Likewise, we can obtain the auxiliary function of *J*(*H*_*U*_) as follows:

$$\begin{array}{c} \begin{array}{c} G\left(H_{U},{H^{\prime }}_{U}\right)=-\left(1+\alpha \right)Tr\left(WV_{U}^{T}H_{U}\right)\\ +\frac{1}{2}\sum_{ij}\frac{{\left(H_{U}^{\prime }WW^{T}\right)}_{ij}}{{\left(H_{U}^{\prime }\right)}_{ij}}{\left(H_{U}\right)}_{ij}^{2}+\frac{\alpha }{2}\sum_{ij}\frac{{\left(V_{U}V_{U}^{T}H_{U}^{\prime }\right)}_{ij}}{{\left(H_{U}^{\prime }\right)}_{ij}}{\left(H_{U}\right)}_{ij}^{2}\\ +\frac{\beta }{2}Tr\left(H_{U}^{T}A-2W^{T}H_{U}^{T}V_{U}+BH_{U}^{T}\right), \end{array} \end{array}$$ (13)

Setting $\frac{\partial G(H_{U},H_{U}^{\prime })}{\partial {\left(H_{U}\right)}_{ij}}=0$, we have

$$\begin{array}{c} {\left(H_{U}\right)}_{ij}={\left(H_{U}^{\prime }\right)}_{ij}\frac{\left(1+\alpha +\beta \right){\left(V_{U}W^{T}\right)}_{ij}}{{\left({H^{\prime }}_{U}WW^{T}+\alpha V_{U}V_{U}^{T}{H^{\prime }}_{U}+\beta /2\left(A+B\right)\right)}_{ij}}, \end{array}$$ (14)

Thus, according to Eq (14), we also obtain the update rule (7) for *H*_*U*_.

Moreover, according to Eqs (10), (12) and (14), we have

$$\begin{array}{c} J\left(W^{t+1},H_{U}^{t+1}\right)\leq J\left(W^{t+1},H_{U}^{t}\right)\leq J\left(W^{t},H_{U}^{t}\right). \end{array}$$ (15)

Based on Eq (15), these update rules always guarantee that the objective function monotonically decreases. Thus, this completes the proof. ■

According to the above theorem, we summarize the multiplicative update rule (MUR) for Semi-PNMF in **Algorithm 1**.

**Algorithm 1** MUR for Semi-PNMF

**Input**: Examples *V* ∈ *R* ^*m* × *n*^, penalty parameter *α*, partial label matrix *Q*.

**Output**: *H*_*U*_.

 1: Randomly initialize *W* ^0^ and $H_{U}^{0}$, and *l* = 0.

 2: **repeat**

 3:  Update *W*_*l*+1_ according to Eq (6).

 4:  Calculate $H_{U}^{l+1}$ according to Eq (7).

 5:  *l* ← *l*+1.

 6: **until** {Stopping criterion $\frac{{∥J^{l+1}-J^{l}∥}_{F}}{{∥J^{l}∥}_{F}}<ɛ$ is satisfied.}

 7: $H_{U}=H_{U}^{l}$.

To reduce the time overhead, **Algorithm 1** utilizes the objective relative error as the stopping criterion; in addition, set *ɛ* to 10^−7^ in our experiments. The main time cost of **Algorithm 1** lies in line 3 and line 4. Their time complexities are *O*(*r* ^2^ *n*+*mrn*+*r* ^2^ *m*+*rm*) and *O*(*mr*(*n* − *d*)+*r* ^2^ *m*+*rm*+*r* ^2^+*r* ^2^(*n* − *d*)), respectively. Thus, the total time complexity of **Algorithm 1** is *O*(*r* ^2^ *n*+*mrn*+*mr*(*n* − *d*)+*mrd*+*r* ^2^ *m*+*rm*+*r* ^2^+*r* ^2^(*n* − *d*)).

## Results

This section conducts a series of experiments on both synthetic and real-world datasets to verify the method proposed in this paper.

### Synthetic Dataset

This section generates a small synthetic dataset to clarify the mechanism of Semi-PNMF. The synthetic dataset consists of three categories constructed by the following random samples:

$$\begin{array}{c} y_{1}={\left[1,0.8,0.8\right]}^{T}+0.1x, \end{array}$$

$$\begin{array}{c} y_{2}={\left[0.8,0.8,0.8\right]}^{T}+0.1x, \end{array}$$

and

$$\begin{array}{c} y_{3}={\left[0.8,1,0.7\right]}^{T}+0.15x, \end{array}$$

where *x* ∈ *R* ^3^, and each of its entry is sampled from the standard uniform distribution *U*(0,1). For each category, we randomly generated 10 samples, within which three samples were selected as labeled samples and the rest as unlabeled ones. Therefore, the synthetic dataset contains 30 samples in total. For clear illustration, three categories are marked as three different colors, and the labeled and unlabeled samples are distinguished by two shapes.

Fig 1(a) and 1(b) shows the ground truth and resultant labeled results of the unlabeled samples by Semi-PNMF, respectively, while Fig 1(c) and 1(d) displays the learned coefficients of the unlabeled samples and basis. In Fig 1(d), each row of the learned basis has different colors, implying that the basis stands for the centroids of different categories and owns the discriminative representation ability. According to Fig 1(c), each row of the learned coefficients is the lower-dimensional coefficient of the corresponding unlabeled sample. The larger the entry of the coefficient is, the darker its color is. As shown in Fig 1(c), the maximum entry of the coefficient largely exceeds the other entries. All maximum entries make the coefficients take up the diagonal form and imply the cluster memberships of all the samples. Thus, it is reasonable to select the index of the maximum entry of the coefficient as the classes of an unlabeled sample. This verifies our previous intuition. Since all samples shares the common basis, their coefficients become close to each other if they have the same labels. We impose the restriction that the coefficients of labeled samples be equivalent to their label vectors, and thus this also induces the coefficients of the unlabeled to be close to their label vectors. In this way, Semi-PNMF can propagate the labels of the labeled samples to the unlabeled ones. The propagation procedure is illustrated in Fig 2.

### GCM Dataset

This experiment merely compares traditional semi-supervised learning methods including low density separation (LDS, [14]), transductive SVM (TSVM, [16]), constrained NMF (CNMF, [24]), soft-constrained NMF (SCNMF, [25]) and Semi-PNMF by separating different types of cancers on the GCM dataset. The GCM dataset [8] contains the expression profiles of 218 tumor samples representing 14 common human cancer classes. It is available on the public website: http://www.broadinstitute.org/cgi-bin/cancer/datasets.cgi, and can also be downloaded from the website: https://zenodo.org/record/21712. According to [8], we combine the training and testing set of this gene expression data into a dataset for cancer classification. Thus, the combined dataset contains 198 samples with 16,063 genes. Table 1 gives a brief description of this dataset. To remove very low noisy values and saturation effects of very high values, we bound the gene expression data into a specific box constraint ranging from 20 to 16,000 units and then exclude those genes whose ratios and absolute variations across samples are under 5 and 500, respectively. Consequently, the resultant expression profile dataset contains the 11,370 genes passing. We compare the effectiveness of Semi-PNMF with LDS, TSVM, CNMF and SCNMF under varying configurations. Both CNMF and SCNMF involve no parameter tuning. For Semi-PNMF, we set two parameters *α* = 2, and *β* = 0.0001, respectively. Because these representative methods enable convergence within 1,500 iteration rounds, we set the maximum number of loops to 1,500. For LDS and TSVM, we adopt the parameter settings provided in the source code to obtain the classification results.

**Table 1:** Description of the GCM dataset.

Cancer Types	Number of Samples
Breast adenocarcinoma (BR)	12
Prostate adenocarcinoma (PR)	14
Lung adenocarcinoma (LU)	12
Colorectal adenocarcinoma (CO)	12
Lymphoma (LY)	22
Bladder transitional cell carcinoma (BL)	11
Melanoma (ML)	10
Uterus adenocarcinoma (UT)	10
Leukemia (LE)	30
Renal cell carcinoma (RE)	11
Pancreas adenocarcinoma (PA)	11
Ovarlan adenocarcinoma (OV)	12
Pleural mesothelioma (MS)	11
Central nervous system (CNS)	20
Total	198

**Fig 3:**
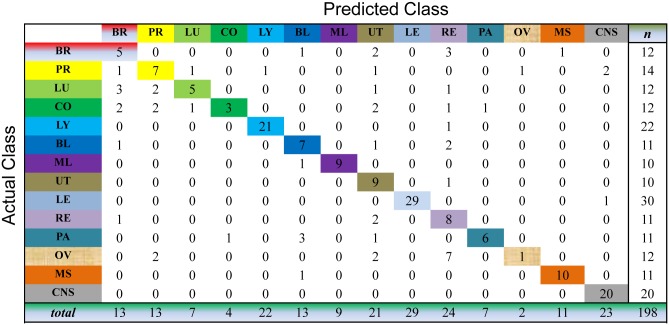
Confusion matrix of prediction results using Semi-PNMF, which achieves a total accuracy of 70.71%. Matrix delineates distribution of actual compared with predicted class membership for multiclass cancer prediction on the GCM dataset.

**Fig 4:**
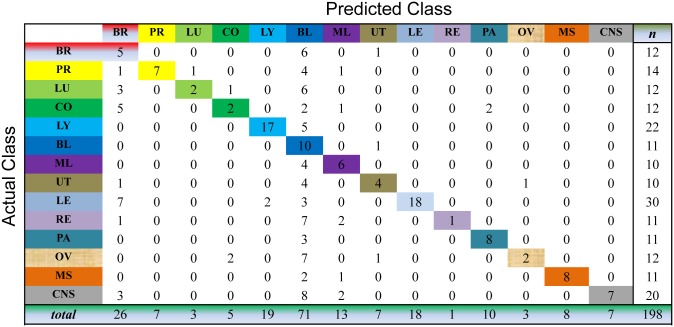
Confusion matrix of prediction results using SCNMF, which achieves a total accuracy of 48.99%. Matrix delineates distribution of actual compared with predicted class membership for multiclass cancer prediction on the GCM dataset.

**Fig 5:**
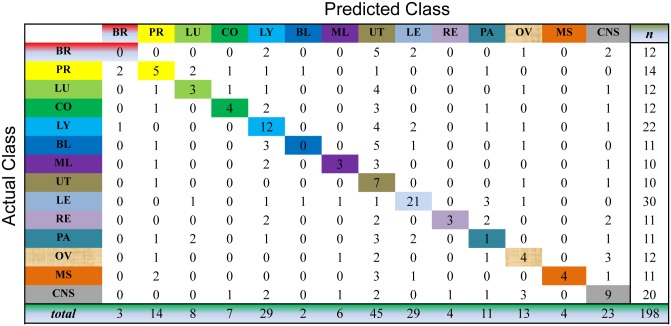
Confusion matrix of prediction results using CNMF, which achieves a total accuracy of 38.4%. Matrix delineates distribution of actual compared with predicted class membership for multiclass cancer prediction on the GCM dataset.

**Fig 6:**
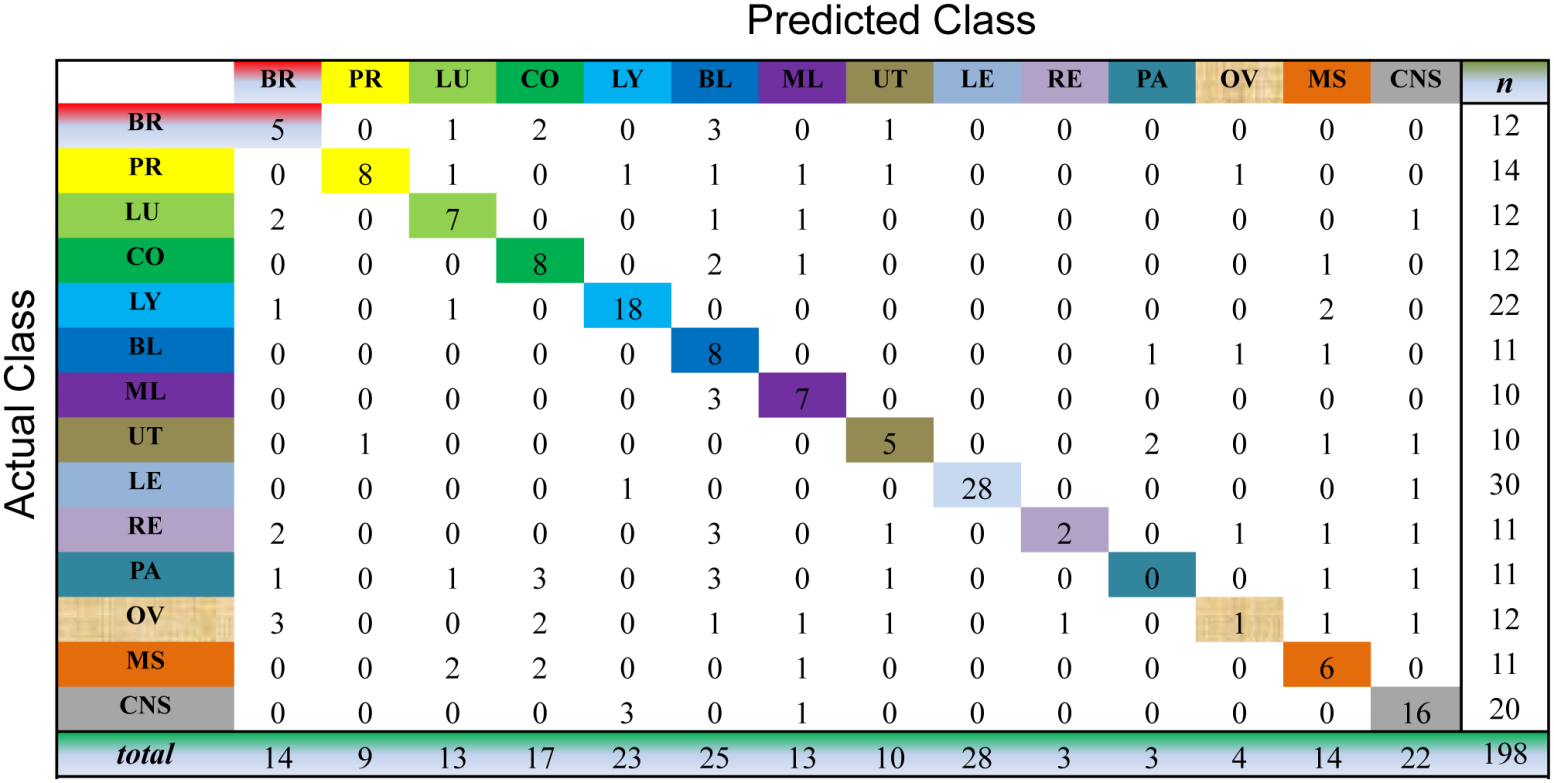
Confusion matrix of prediction results using LDS, which achieves a total accuracy of 60.1%. Matrix delineates distribution of actual compared with predicted class membership for multiclass cancer prediction on the GCM dataset.

**Fig 7:**
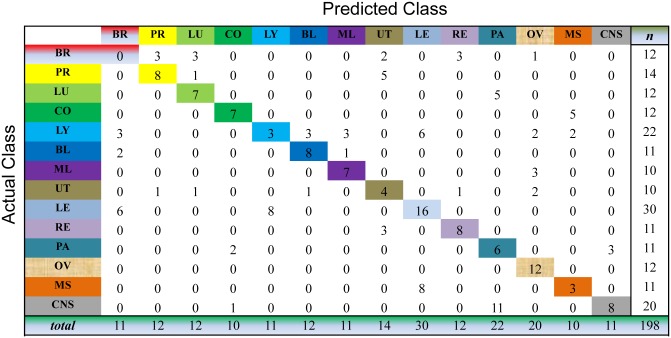
Confusion matrix of prediction results using TSVM, which achieves a total accuracy of 48.99%. Matrix delineates distribution of actual compared with predicted class membership for multiclass cancer prediction on the GCM dataset.

We evaluate the cancer classification by the cross-validation over the whole dataset. This process selects one sample as the unlabeled sample and, meanwhile, learns the prediction model on all the samples for cancer diagnosis. For the unlabeled sample, we choose the index of the largest value of the resultant consensus matrix to predict the classes of this sample. As shown in Figs 3 to 7, the confusion matrix of the predicted results of Semi-PNMF, CNMF, SCNMF, LDS and TSVM are reported in detail. Each column denotes how many the unlabeled samples are assigned to each cancer, while each row signifies the number of the unlabeled samples affiliated to the real tumor type. Each color not only represents a specific cancer type but also highlights the correct prediction results, i.e., the diagonal elements of the confusion matrix.

Figs 3 to 7 imply that Semi-PNMF can identify different tumor types more accurately than the representative methods. For example, when working with two labeled samples from each tumor type, Semi-PNMF achieves 70.71% classification accuracy and exceeds LDS, TSVM, SCNMF, and CNMF by 10.6%, 21.72%, 21.72%, and 32.3%, respectively. Moreover, Table 2 further implies the effectiveness of Semi-PNMF compared with CNMF, SCNMF, TSVM, and LDS in terms of both sensitivity and specificity. For completeness, we list their definitions as follows:

$$\begin{array}{c} sensitivity=\frac{TP}{TP+FN}, \end{array}$$ (16)

and

$$\begin{array}{c} specificity=\frac{TN}{TN+FP}, \end{array}$$ (17)

where *TP*, *TN*, *FP*, and *FN* denote the number of true positive, true negative, false positive and false negative samples, respectively.

**Table 2:** Sensitivity and Specificity of the compared methods over 14 cancer subtypes on the GCM dataset.

	Sensitivity					Specificity				
	CNMF	SCNMF	TSVM	LDS	Semi-PNMF	CNMF	SCNMF	TSVM	LDS	Semi-PNMF
BR	0	**0.42**	0	**0.42**	**0.42**	**0.98**	0.89	0.94	0.95	0.96
PR	0.36	0.5	**0.57**	**0.57**	0.5	0.95	**1**	0.98	0.99	0.98
LU	0.25	0.17	**0.58**	**0.58**	0.42	0.97	**0.99**	0.97	0.97	**0.99**
CO	0.33	0.17	0.58	**0.67**	0.25	0.98	0.98	0.98	0.95	**0.99**
LY	0.55	0.77	0.14	0.82	**0.95**	0.9	**0.99**	0.95	0.97	**0.99**
BL	0	**0.91**	0.73	0.73	0.64	**0.99**	0.67	0.98	0.91	0.97
ML	0.3	0.6	0.7	0.7	**0.9**	0.98	0.96	0.98	0.97	**1**
UT	0.7	0.4	0.4	0.5	**0.9**	0.79	**0.98**	0.95	0.97	0.94
LE	0.7	0.6	0.53	0.93	**0.97**	0.95	**1**	0.92	**1**	**1**
RE	0.27	0.09	0.73	0.18	**0.73**	0.99	**1**	0.98	0.99	0.91
PA	0.09	**0.73**	0.55	0	0.55	0.95	**0.99**	0.91	0.98	**0.99**
OV	0.33	0.17	**1**	0.083	0.08	0.95	**0.99**	0.96	0.98	**0.99**
MS	0.36	0.73	0.27	0.55	**0.91**	**1**	**1**	0.96	0.96	0.99
CNS	0.45	0.35	0.4	0.8	**1**	0.92	**1**	0.98	0.97	0.98
Avg.	0.34	0.47	0.51	0.538	**0.66**	0.95	0.96	0.96	0.97	**0.98**

The number of labeled examples is an important factor affecting the performance of semi-supervised learning methods. Hence, it is very necessary to observe the classification accuracy of Semi-PNMF under different numbers (1–6) of labeled samples in each class. Here, we randomly select different numbers of examples from each class as labeled examples and regard the rest as unlabeled. For fair comparison, we independently conduct 100 individual experiment trails to remove the effect of randomness.

**Fig 8:**
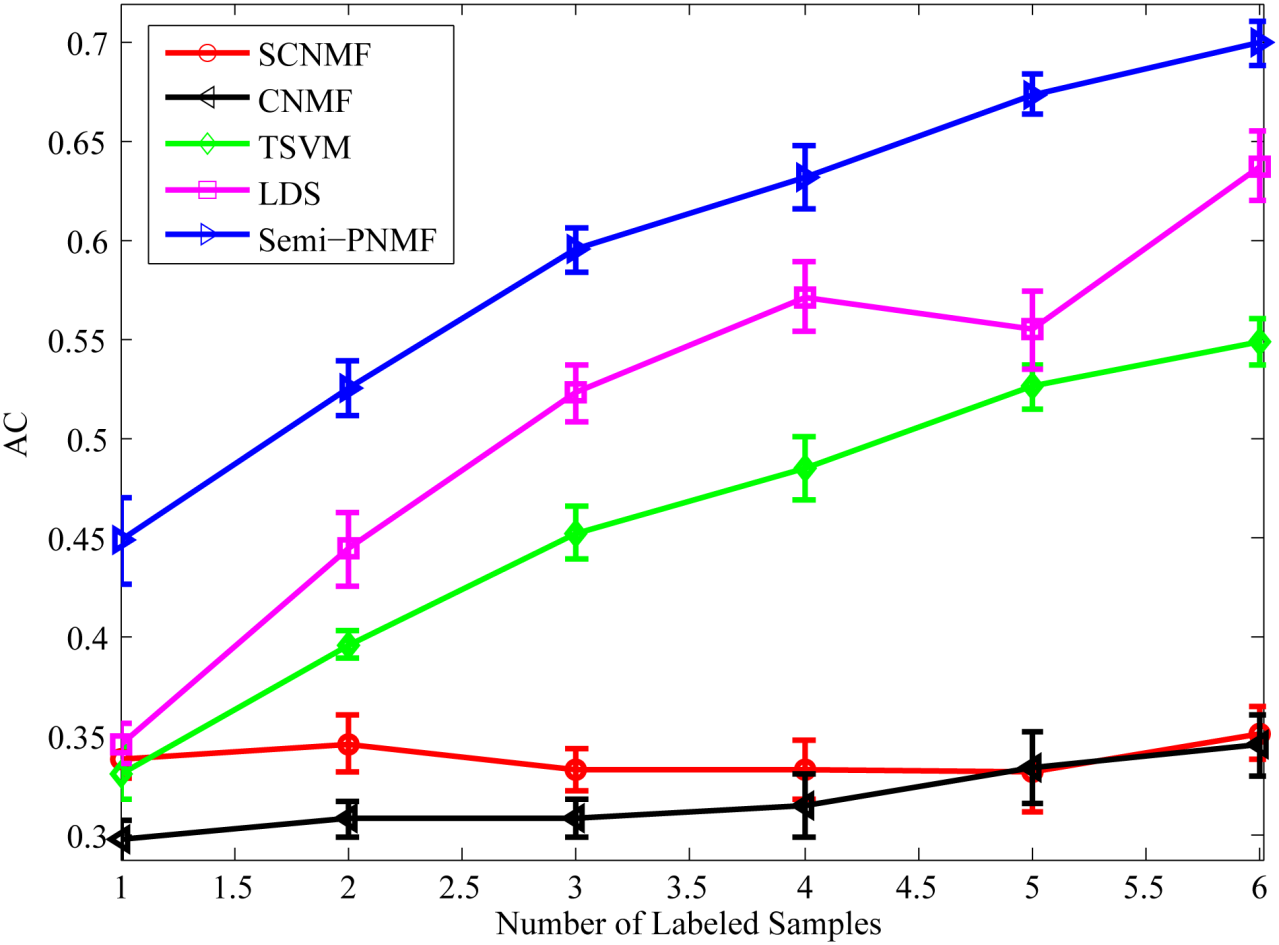
Average accuracies versus different numbers (1–6) of the labeled samples for each class of the GCM dataset.

Fig 8 compares the average accuracy of CNMF, SCNMF, TSVM, LDS, and Semi-PNMF under different numbers of labeled samples for each class. It also shows that Semi-PNMF achieves the highest accuracy and takes on an increasing tendency with the rise in the number of labeled samples.

### Acute Leukemia Dataset

We also conduct a cancer classification experiment to verify the classification performance of Semi-PNMF compared with low density separation (LDS, [14]), transductive SVM (TSVM, [16]), constrained NMF (CNMF, [24]), and soft-constrained NMF (SCNMF, [25]) on another popular dataset, i.e., the Acute Leukemia dataset [36]. This dataset comes from Gene Expression Omnibus (http://www.ncbi.nlm.nih.gov/geo/query/acc.cgi?acc=GSE13159), and can also be downloaded from the website: https://zenodo.org/record/21712. We replace the unavailable entries of this dataset with the average values of their *k*-nearest neighbor elements. This dataset consists of 2,096 samples along with 54,675 probes in total. This dataset contains different cancer subtypes of the acute leukemia and thus is not suited for cancer classification in contrast with the GCM dataset. Table 3 gives a brief description of this dataset. Then, we feed this dataset to all the compared methods.

**Table 3:** Description of the Acute Leukemia dataset.

Cancer Types	Number of Samples
Mature B-ALL with t(8;14)	13
Pro-B-ALL with t(11q23)/MLL	70
c-ALL/pre-B-ALL with t(9;22)	122
T-ALL	174
ALL with t(12;21)	58
ALL with t(1;19)	36
ALL with hyperdiploid karyotype	40
c-ALL/pre-B-ALL without t(9;22)	237
AML with t(8;21)	40
AML with t(15;17)	37
AML with inv(16)/t(16;16)	28
AML with t(11q23)/MLL	38
AML with normal karyotype+other abnormalities	351
AML complex aberrant karyotype	48
CLL	448
CML	76
MDS	206
Non-leukemia and healthy bone marrow	74
Total	2,096

Abbreviations: B-ALL, B-cell acute lymphoblastic leukemia; MLL, myeloid/lymphoid or mixed-lineage leukemia; pre, precursor; c-ALL, childhood acute lymphoblastic leukemia; T-ALL, T-cell acute lymphoblastic leukemia; ALL, acute lymphoblastic leukemia; AML, acute myeloid leukemia; CLL, chronic lymphocytic leukemia; CML, chronic myelogenous leukemia; MDS, myelodysplastic syndrome.

**Fig 9:**
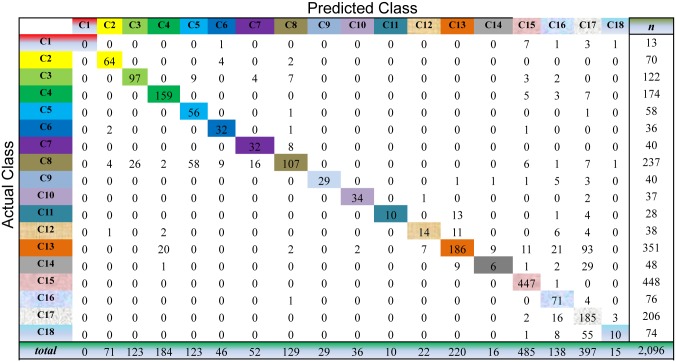
Confusion matrix of prediction results using Semi-PNMF, which achieves a total accuracy of 73.43%. Matrix delineates distribution of actual compared with predicted class membership for multiclass cancer prediction on the Acute Leukemia dataset.

**Fig 10:**
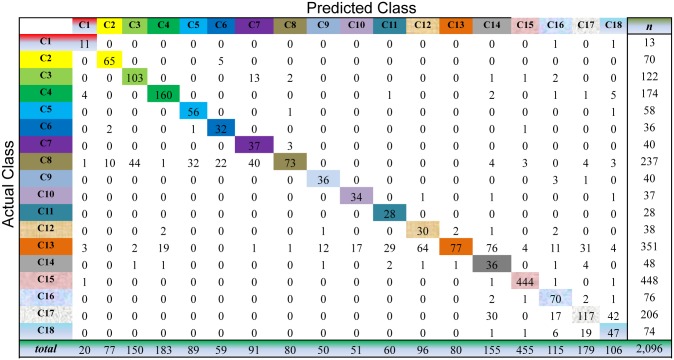
Confusion matrix of prediction results using SCNMF, which achieves a total accuracy of 69.47%. Matrix delineates distribution of actual compared with predicted class membership for multiclass cancer prediction on the Acute Leukemia dataset.

**Fig 11:**
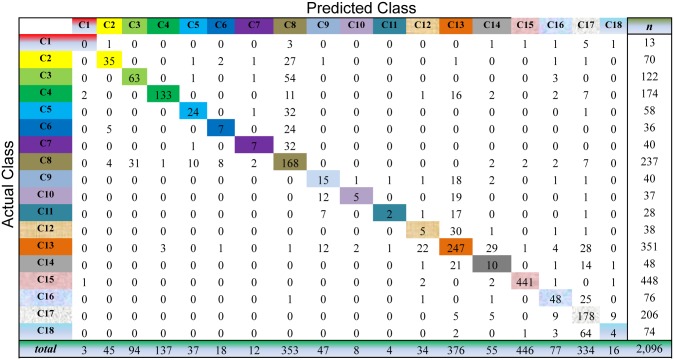
Confusion matrix of prediction results using CNMF, which achieves a total accuracy of 66.41%. Matrix delineates distribution of actual compared with predicted class membership for multiclass cancer prediction on the Acute Leukemia dataset.

**Fig 12:**
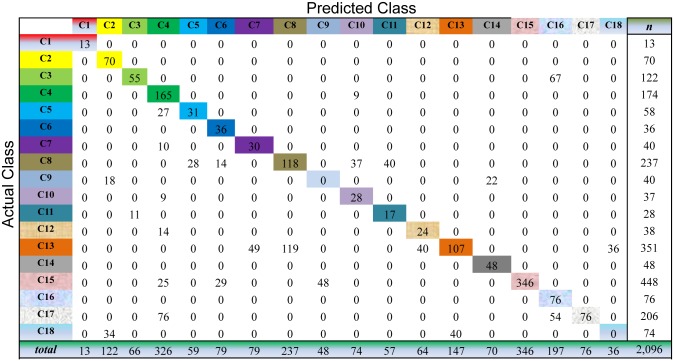
Confusion matrix of prediction results using LDS, which achieves a total accuracy of 59.16%. Matrix delineates distribution of actual compared with predicted class membership for multiclass cancer prediction on the Acute Leukemia dataset.

**Fig 13:**
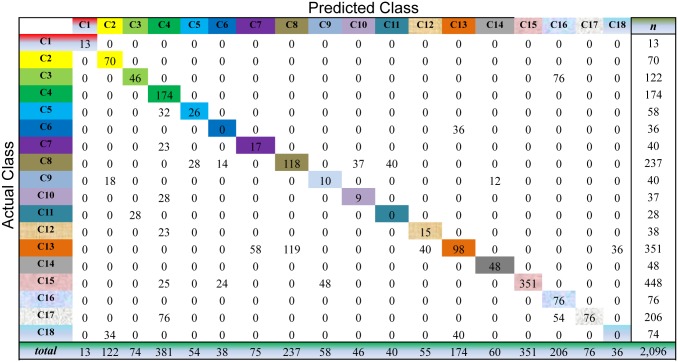
Confusion matrix of prediction results using TSVM, which achieves a total accuracy of 54.72%. Matrix delineates distribution of actual compared with predicted class membership for multiclass cancer prediction on the Acute Leukemia dataset.

For Semi-PNMF, we set two parameters *α* = 0.2, and *β* = 0.01. For the traditional semi-supervised learning methods, we adopt the same configurations as the above subsection. The cross-validation process of the above subsection is repeatedly conducted to evaluate the compared methods on this dataset. As shown in Figs 9 to 13, the confusion matrix of the predicted results of Semi-PNMF, CNMF, SCNMF, LDS and TSVM are reported in detail. Each column denotes how many unlabeled samples are assigned to each cancer subtype, while each row signifies the number of unlabeled samples affiliated to the real tumor subtype. Each color not only represents a specific cancer subtype but also highlights the correct prediction results, i.e., the diagonal elements of the confusion matrix.

Figs 9 to 13 imply that Semi-PNMF can identify different tumor types more accurately than the representative methods. Semi-PNMF achieves the highest total classification accuracy compared with CNMF, SCNMF, TSVM and LDS in terms of the prediction results in the confusion matrix. Moreover, Table 4 also indicates that Semi-PNMF consistently outperforms the compared methods on eighteen cancer subtypes in terms of both sensitivity and specificity. In summary, these results suggest the effectiveness of Semi-PNMF in cancer classification.

**Table 4:** Sensitivity and Specificity of the compared methods over 18 cancer subtypes on the Acute Leukemia dataset.

	Sensitivity					Specificity				
	CNMF	SCNMF	TSVM	LDS	Semi-PNMF	CNMF	SCNMF	TSVM	LDS	Semi-PNMF
C1	0	0.85	**1**	**1**	0	**1**	**1**	**1**	**1**	**1**
C2	0.5	0.93	**1**	**1**	0.91	**1**	0.99	0.97	0.97	**1**
C3	0.52	**0.84**	0.38	0.45	0.79	0.98	0.98	**0.99**	**0.99**	**0.99**
C4	0.76	0.92	**1**	0.95	0.91	**1**	0.99	0.89	0.92	0.97
C5	0.41	**0.97**	0.45	0.53	**0.97**	**0.99**	0.98	**0.99**	**0.99**	**0.99**
C6	0.19	0.89	0	**1**	0.89	**0.99**	**0.99**	0.98	0.98	**0.99**
C7	0.18	**0.93**	0.43	0.75	0.8	**1**	0.97	0.97	0.97	0.99
C8	**0.71**	0.31	0.5	0.5	0.45	0.9	1	0.94	0.94	**1**
C9	0.38	**0.9**	0.25	0	0.73	0.98	0.99	0.98	0.98	**1**
C10	0.14	**0.92**	0.24	0.76	**0.92**	**1**	0.99	0.98	0.98	**1**
C11	0.07	**1**	0	0.61	0.36	**1**	0.98	0.98	0.98	**1**
C12	0.13	**0.79**	0.39	0.63	0.37	**0.99**	0.98	0.98	0.98	0.98
C13	**0.7**	0.22	0.28	0.3	0.53	0.93	1	0.96	0.98	**1**
C14	0.21	0.75	**1**	**1**	0.13	0.98	0.94	**0.99**	**0.99**	0.98
C15	0.98	0.99	0.78	0.77	**1**	**1**	0.99	**1**	**1**	0.97
C16	0.63	0.92	**1**	**1**	0.93	0.99	0.98	0.94	0.94	0.97
C17	0.86	0.57	0.37	0.37	**0.9**	0.92	0.97	**1**	**1**	0.89
C18	0.05	**0.64**	0	0	0.14	0.99	0.97	0.98	0.98	**1**
Avg.	0.41	**0.8**	0.5	0.646	0.65	0.98	0.9828	0.9733	0.9761	**0.9844**

Each row indicates the specific cancer sub-style corresponding to each row of Table 3.

The number of the labeled samples is an important factor affecting the performance of semi-supervised learning methods. Hence, it is very necessary to observe the classification accuracy of Semi-PNMF under different numbers (1–6) of labeled samples in each class. Here, we randomly select different numbers of examples from each class as labeled examples and regard the rest as unlabeled. Then, we independently conduct 10 individual experiment trails to remove the effect of randomness.

**Fig 14:**
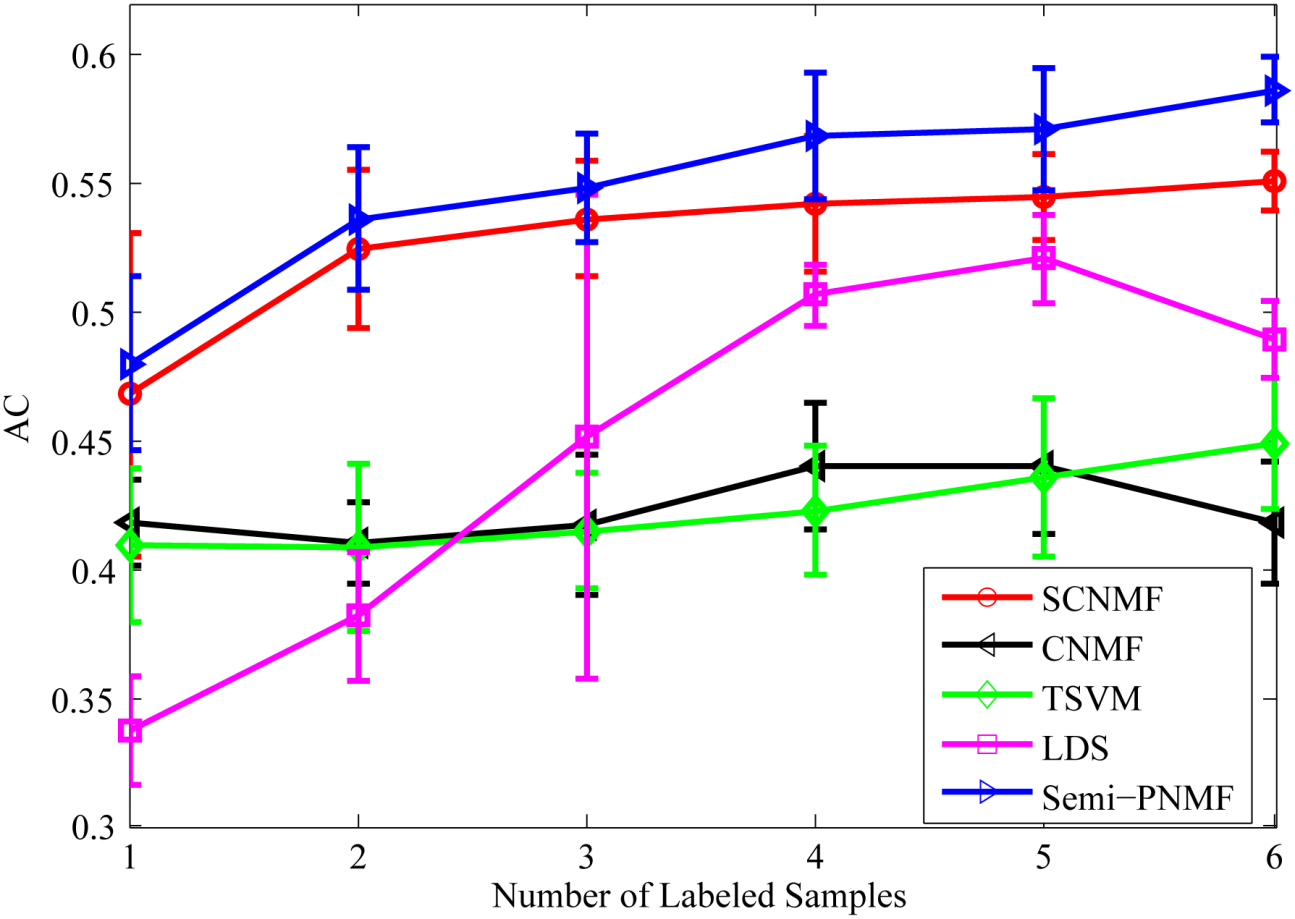
Average accuracies versus different numbers (1–6) of the labeled samples for each class on the Acute Leukemia dataset.

Fig 14 compares the average accuracy of CNMF, SCNMF, TSVM, LDS, and Semi-PNMF under different numbers of labeled samples for each class. It also shows that Semi-PNMF achieves the highest accuracy and has an increasing tendency with the rise in the number of labeled samples.

## Discussion

This paper proposes the semi-supervised PNMF method (Semi-PNMF), which incorporates two types of constraints as well as the auxiliary basis to boost PNMF. Particularly, Semi-PNMF utilizes the linear combination of examples to approximate the cluster centroids such that the cluster centroids have more powerful representative ability. To effectively indicate the classes of unlabeled samples, Semi-PNMF enforces the coefficients of labeled samples to approach their labels, meanwhile representing the unlabeled samples using the identical cluster centroid. To optimize Semi-PNMF, we devised the multiplicative update rule (MUR) to establish the convergence guarantee. Experiments of cancer classification on two real-world datasets show that Semi-PNMF outperforms the representative methods in terms of quantity.

Recently, Bayesian methods that incorporate both sparsity and a large number of covariates in the model have been extensively used for parameter estimation and classification in data sets compared to small sample sizes such as gene expression data [39–41]. They also improve model accuracy by introducing a slight bias in the model [40]. In future works, we can borrow from the merits of Bayesian methods to further improve the classification performance of Semi-PNMF for a large-scale dataset. Semi-PNMF has provided a flexible framework for learning methods in cancer data processing and can be utilized in other applications such as cancer recurrence [42, 43].
